# Characteristics of gut microbiota and fecal metabolomes in patients with celiac disease in Northwest China

**DOI:** 10.3389/fmicb.2022.1020977

**Published:** 2022-11-28

**Authors:** Tian Shi, Yan Feng, Weidong Liu, Huan Liu, Ting Li, Man Wang, Ziqiong Li, Jiajie Lu, Adilai Abudurexiti, Ayinuer Maimaitireyimu, Jiali Hu, Feng Gao

**Affiliations:** ^1^Department of Gastroenterology, People’s Hospital of Xinjiang Uygur Autonomous Region, Urumqi, China; ^2^Xinjiang Clinical Research Center for Digestive Diseases, Urumqi, China

**Keywords:** celiac disease, gut microbiota, fecal metabolomics, correlation analysis, dietary habits, genetic background, Northwest China

## Abstract

Celiac disease (CD) is an autoimmune small bowel disease. The pattern of gut microbiota is closely related to dietary habits, genetic background, and geographical factors. There is a lack of research on CD-related gut microbiota in China. This study aimed to use 16S rDNA sequencing and metabolomics to analyze the fecal microbial composition and metabolome characteristics in patients diagnosed with CD in Northwest China, and to screen potential biomarkers that could be used for its diagnosis. A significant difference in the gut microbiota composition was observed between the CD and healthy controls groups. At the genus level, the abundance of *Streptococcus*, *Lactobacillus*, *Veillonella*, and *Allisonella* communities in the CD group were increased (*Q* < 0.05). Furthermore, the abundance of *Ruminococcus*, *Faecalibacterium*, *Blautia*, *Gemmiger*, and *Anaerostipes* community in this group were decreased (*Q* < 0.05). A total of 222 different fecal metabolites were identified in the two groups, suggesting that CD patients have a one-carbon metabolism defect. Four species of bacteria and six metabolites were selected as potential biomarkers using a random forest model. Correlation analysis showed that changes in the gut microbiota were significantly correlated with changes in fecal metabolite levels. In conclusion, the patterns of distribution of gut microbiota and metabolomics in patients with CD in Northwest China were found to be unique to these individuals. This has opened up a new way to explore potential beneficial effects of supplementing specific nutrients and potential diagnostic and therapeutic targets in the future.

## Introduction

Celiac disease (CD) is an autoimmune inflammatory disease affecting the small intestine, induced by the ingestion of gluten in genetically susceptible individuals (Ludvigsson et al., 2013; Lebwohl et al., 2018). The pooled global prevalence of biopsy-confirmed CD is 0.7%, and the pooled global prevalence of positive results from serologic testing is 1.4% (Singh et al., 2018). This disease can develop at any age, including geriatric populations (Collin et al., 2018). In recent years, the incidence rate of CD has been increasing, partly owing to improved understanding and detection of the disease and to the effective increase in the prevalence of immune disease (Lebwohl and Rubio-Tapia, 2021). The occurrence and development of CD is the result of the interaction of genetic, immune, and environmental factors (D’Avino et al., 2021). Gluten is recognized as an external trigger for CD (Serena et al., 2019b). Gluten is a protein complex mainly existing in wheat, barley, and rye (Wu et al., 2021). Due to its richness in glutamine and proline, it is highly resistant to protease hydrolysis. As a result, it cannot be fully digested by human digestive enzymes, resulting in large peptides (such as 33-mer) that can accumulate on the lamina propria of the small intestine; this can trigger abnormal immune responses in CD susceptible individuals (carrying HLA-DQ2/DQ8 genes), damage the intestinal mucosa, and lead to the occurrence of CD (Shan et al., 2002; Molberg et al., 2005; Zhu et al., 2022). Approximately 30% of the general population carries the HLA-DQ2/8 susceptibility gene, but only 1% of these individuals will develop CD. This indicates that there are other factors involved in the development of the disease, such as infant delivery style, feeding type, timing and amount of gluten intake, and infection and early antibiotic exposure, which indirectly play a pathogenic role in regulating the gut microbiota (Akobeng et al., 2020).

Gut microbiota play an important role in host nutrition metabolism, maintaining the structural integrity of the intestinal mucosal barrier, immune regulation, and resistance to pathogens (Thursby and Juge, 2017; Rinninella et al., 2019). A growing body of evidence indicates that the occurrence of CD is closely related to the ecological imbalance of gut microbiota (Valitutti et al., 2019). Many host-related factors—such as age, sex, genetic susceptibility, and health status—and environmental factors influence the composition of gut microbiota (Olivares et al., 2015; Rehman et al., 2016; Valitutti et al., 2019; Trakman et al., 2022). Garcia-Mazcorro et al. believed that the gut microbiota distribution pattern and metabolic activities differ significantly from person to person, and the impact of food composition, dietary habits and economic level on health varies greatly in between and within countries (Garcia-Mazcorro et al., 2018a; Rinninella et al., 2019). It is suggested that more studies based on local populations should be conducted to draw useful conclusions in biology and medicine, which will pave the way for discovering better treatment protocols for patients with CD.

The gut microbiota also acts as a metabolic organ, interacting with host cells and providing functional support needed to maintain homeostasis (Olshan et al., 2020). Metabolomics is an important part of systems biology and is one of the key technologies for understanding the function of these microbial metabolites (Bauermeister et al., 2022). The fecal metabolome provides functional readings of microbial activity and can serve as an intermediate phenotype to mediate these interactions (Zierer et al., 2018). The metabolic capacity of the gut microbiota has been studied in the CD population, and there are differences in short-chain fatty acid metabolism between untreated and treated CD patients and healthy controls (HC) (Nistal et al., 2012b), as well as differences in specific microbiota that metabolize gluten in CD patients (Caminero et al., 2015). Thus, differences in microbial metabolites in CD patients as compared to the normal population indicate the functional role of the microbiota in the pathogenesis of CD. However, the specific mode of action of gut microbiota disorders involved in the occurrence of CD is not clear; it is likely to cause changes in relevant terminal metabolites in the feces of patients. At present, there is still a lack of consensus on specific bacteria and metabolites in patients with CD, and the identification of specific CD metabolome phenotypes could help identify additional diagnostic tools and therapeutic interventions.

A meta-analysis showed that the seroprevalence of CD in the general Chinese population is 0.27%, and varies with the geographical origin of the patients, being higher in northern China than in southern China (Zhou et al., 2021). Xinjiang, located in the Northwest of China, has a population of 25.85 million people divided between 55 ethnic groups. Some major ethnic groups are Uyghur (45%), Han (42%), and Kazakh (7%) (Statistics Bureau of Xinjiang Uygur Autonomous Region, 2021). Wheat is the primary food crop for this population. Furthermore, historically, they are characterized by different degrees of genetic exchange with western Europeans (Caucasians). Zhou et al. (2020) reported that the detection rate of CD in Xinjiang is approximately 1.27%, which is higher than the reported national average. These factors may partially account for this high prevalence, although additional external factors should also be taken into consideration. Xinjiang has a unique geographical environment, genetic background, and population composition, which results in the special living and eating habits of its residents. It is well known that differences in dietary habits and genetic background may lead to differences in gut microbiota. Therefore, the current research results of other countries and regions cannot be extrapolated to the characteristics of CD in Xinjiang. The purpose of this study is to analyze the fecal microbial composition and metabolome characteristics in patients diagnosed with CD in Northwest China, and to screen potential biomarkers which could be used for diagnosing the disease. To the best of our knowledge, this study is the first in China to investigate gut microbiota in this clinically important condition.

## Materials and methods

### Recruitment of participants

We recruited 30 patients (≥18 years old) who were initially diagnosed with CD in the People’s Hospital of Xinjiang Uygur Autonomous Region from July 2020 to December 2021 (see Supplementary Table S1 for details). Simultaneously, 30 age-, sex-and ethnic-matched healthy subjects were recruited. All patients in the HC group were symptom-free volunteers with normal diets and free of a recent or chronic illness. Furthermore, they were screened using serum-specific antibodise (anti-tissue transglutaminase antibody and anti-endomysial antibody) to exclude CD (see Supplementary Table S2 for details). The following exclusion criteria were applied to all groups:

1. Patients with parasitic diseases, intestinal infections, irritable bowel syndrome, gastrointestinal dysfunction, and other organic gastrointestinal diseases (hemorrhage, perforation, malignant tumor, inflammatory bowel disease).
2. Patients with a history of chronic systemic autoimmune diseases with gastrointestinal involvement.
3. Patients who had undergone gastrointestinal resection.
4. Pregnant and lactating women.
5. People who had recently (within the past 4 weeks) used drugs (such as probiotics and prebiotics, proton pump inhibitors, antibiotics, non-steroidal anti-inflammatory drugs, opioids, steroids, laxatives, or antidiarrheal), which might affect the intestinal function and/or microbial communities of the gastrointestinal tract.
6. Patients with a history of food or other allergies within the previous month.
7. Patients unwilling to participate in this study.

Informed consent was obtained from all the participants, and the study was approved by the Ethics Committee of the People’s Hospital of Xinjiang Uygur Autonomous Region (KY20220311067).

### Diagnostic criteria

The diagnostic criteria of CD were as per the 2017 World Gastroenterology Organization global guidelines on Celiac Disease (Bai and Ciacci, 2017). The diagnosis of CD hinges on the presence of positive CD–specific autoantibodies and concomitant diagnostic intestinal biopsies.

### DNA extraction, 16S rDNA sequencing, and data processing

The genomic DNA of fecal samples was extracted by FastDNA Spin Kit for soil (MP bio, United States). The purity and integrity of extracted genomic DNA were detected by 1% agarose gel electrophoresis. The concentration and purity of genomic DNA were measured by Nanodrop 2000 ultraviolet visible spectrophotometer (Thermo Fisher Scientific, USA). For each sample, primers 341F (5′-CCTACGGGNGGCWG CAG-3′) and 805R (5′-GACTACHVGGGTATCTAATCC-3′) were used to amplify the V3-V4 hypervariable region of the 16S rRNA gene. The purified PCR products were used for the construction of an amplification library, which was sequenced by Illumina Miseqbenchtop Sequencer (Illumina, USA) using a 2 × 250-bp-paired end sequencing strategy. The raw read sequences were processed in QIIME2 (Bolyen et al., 2019). Adapter and primer sequences were trimmed by using the cutadapt plugin of QIIME2 and trimmed reads were pooled as a Fasta.gz file format for further analysis in DADA2 (v 1.6.0) pipeline (Callahan et al., 2016). The DADA2 plugin of QIIME2 software was used for data quality filtering, noise reduction, and splicing; the chimera sequences were then removed. Finally, the amplicon sequence variants (ASVs) were obtained.

Taxonomic assignments of ASV representative sequences were compared with the Ribosomal Database Project (RDP) (version 11.5) database by RDP classifier algorithm (Wang et al., 2007) with a confidence threshold of 0.8. The abundance and diversity of species in each group were assessed by the alpha diversity analysis. Chao1, observed species and ACE were calculated to analyze the richness, while Shanno, Simpson, and Coverage were calculated to analyze the diversity. These were calculated using the vegan package (v2.5.6) and visualized by the ggplot2 package (v3.3.0) in the R project.

The beta diversity measured the difference in ASV composition between different samples and was assessed using principal component analysis (PCA) and principal coordinate analysis (PCoA), which were supervised analyses suitable for high dimensional data. Analysis of similarities (ANOSIM) is a function in the vegan package (v2.5.6) used to calculate significance of the beta diversity. The microbiota features in HCs were compared to those in patients with CD using Metastats. PICRUSt2 (v2.3.0) analysis was used to investigate the changes of Kyoto Encyclopedia of Genes and Genomes (KEGG) pathway caused by changes in gut microbiota composition.

### Faecal metabolome profiling and data preprocessing

Liquid chromatography mass spectrometry (LC–MS) was used to detect the non-targeted metabolome in fecal samples. A stool sample of 100 mg was thawed at room temperature, 600 μl methanol (including L-2-chloro-L-phenylalanine, 4 ppm) was added, and the feces were oscillated for 30 s. Then, 100 mg glass beads were added and placed into a tissue grinder, ground at 60 Hz for 90 s, ultrasonic at room temperature for 10 min, and centrifuged at 12,000 rpm at 4°C for 10 min. Samples of 20 μl were extracted from each intestinal sample and mixed to make a quality control sample, and the remaining ones were detected by LC–MS. The samples were separated by ACQUITY UPLC® HSS T3 (2.1 × 150 mm, 1.8 μm) (Waters, Milford, MA, United States) and subjected to mass spectrometry at a column temperature of 40°C and a flow rate of 0.25 ml/min. MS data were obtained using a Thermo Q Exactive Mass Spectrometer (Thermo Fisher Scientific, USA) equipped with an electrospray ionization (ESI) source operating in either positive (ESI +) or negative (ESI -) ion mode.

The raw data were firstly converted to mzXML format by MSConvert in the ProteoWizard software package (v3.0.8789) and processed using XCMS for feature detection, retention time correction and alignment. The metabolites were identified by accuracy mass (<30 ppm) and MS/MS data, which were matched with HMDB,^1^ massbank,^2^ LipidMaps,^3^ mzclound,^4^ and KEGG.^5^ Then, we used orthogonal projections to latent structures discriminant analysis (OPLS-DA) or discriminant analysis model (PLS-DA) to look at differences between groups. To avoid the risk of overfitting, the model parameters R2 and Q2 were computed to evaluate the interpretability and predictability of the models. Variable importance in the projection (VIP) was calculated in the OPLS-DA model. Values of *P* were estimated with paired Student’s t-test on single-dimensional statistical analysis. *p* < 0.05 and VIP > 1 were used as screening conditions for related differential metabolites. The Kyoto Encyclopedia of Genes and Genomes (KEGG)^6^ was used to annotate the metabolites. MetaboAnalyst^7^ database was used for pathway analysis.

### Statistical analysis

Statistical analysis was conducted using SPSS 22.0 (SPSS, Inc., Chicago, IL). Quantitative data that conformed to the normal distribution are presented as mean ± standard deviation, and a t-test was used to compare the groups. Quantitative data with a non-normal distribution are presented as median (Q1, Q3), and the Mann–Whitney U test was used to compare groups. Categorical variables are expressed as frequencies and percentages. Statistical significance was set at *p* < 0.05. The microbiota features in HCs were compared to those in patients with CD using Metastats. *p*-values or *Q*-values (false discovery rate adjusted) <0.05 were considered to be significantly different. *p-*value and VIP value were used to analyze the differential expression of metabolites. *p* < 0.05 and VIP > 1 were considered as potential biomarkers. All significantly altered metabolites or bacterial species were included in the models as potential biomarkers for building a random forest model to distinguish disease status. The receiver operating characteristic (ROC) was analyzed using the R package (plotROC package), and the area under the curve (AUC) was calculated, to illustrate performances of prediction models. The correlation analysis of differential metabolites and differential microbiota was performed using Pearson correlation analysis, and the two omics analysis was performed at the microbial genus level.

## Results

### Baseline characteristics of study participants

The final recruitment included 30 newly diagnosed CD patients and 30 HCs with similar age and lifestyle habits. The average age of the CD group was 40.87 ± 12.35 years, and that of HC group was 39.83 ± 11.19 years. There were no significant differences in age, sex and ethnicity between the two groups (*p* > 0.05), indicating that the two groups were comparable. Among these CD patients, 4 Marsh 3a, 11 Marsh 3b, and 15 Marsh 3c were identified. All HC group patients were screened for serum tTG-IgA antibody and total IgA antibody, and CD was excluded. The demographic characteristics of patients in the CD and HC groups were shown in Table 1. Each fecal sample was sequenced by 16S rDNA sequencing to analyze microbial community composition, diversity and functional potential. Untargeted metabolome detection was performed on all fecal samples using LC–MS to analyze and capture many known and uncharacterized metabolites, including those of potential microbial origin.

**Table 1:** Baseline Characteristics of CD and HC group.

**Characteristic**		**CD (*n* = 30)**	**HC (*n* = 30)**
Sex (*n*, %)	Male	7 (23.3)	7 (23.3)
	Female	23 (76.7)	23 (76.7)
Age (years)	(mean ± standard)	40.87 ± 12.35	39.83 ± 11.19
Ethnic (*n*, %)	Han	4 (13.3)	4 (13.3)
	Uyghur	14 (46.7)	14 (46.7)
	Kazakh	12 (40.0)	12 (40.0)
Marsh grade (*n*, %)	Marsh 3a	4 (13.3)	–
	Marsh 3b	11 (36.7)	–
	Marsh 3c	15 (50.0)	–

### Analysis of gut microbiota diversity

**Figure 1:**
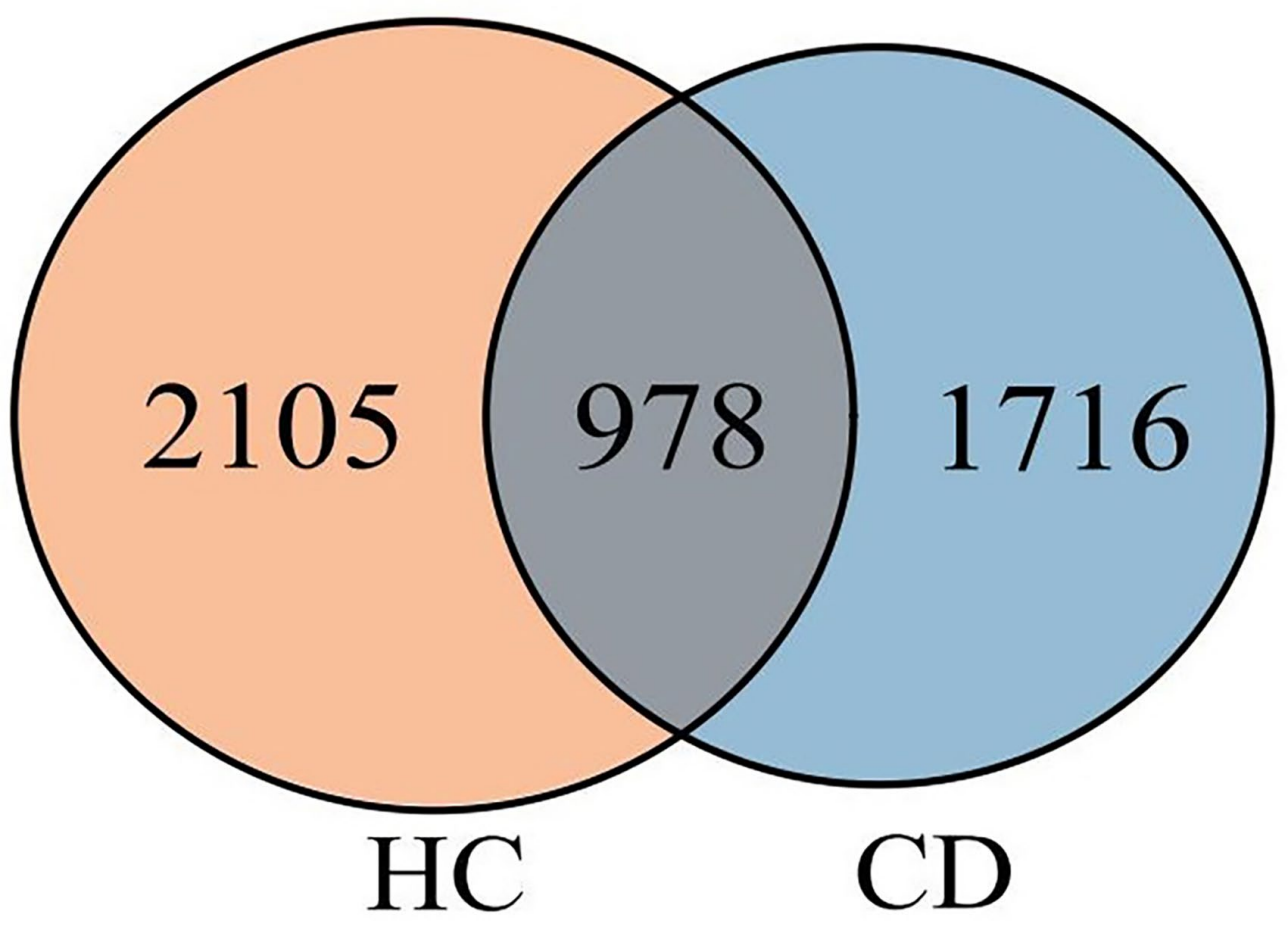
Venn diagram of AVSs from the CD and HC groups.

A total of 4, 024, 566 high-quality reads were obtained from 60 stool samples. Furthermore, a total of 4,799 ASVs were obtained. Of these ASVs, 978 were shared among the three groups, while 2,694 and 3,083 ASVs were specific to the CD and HC groups, respectively (Figure 1).

**Figure 2:**
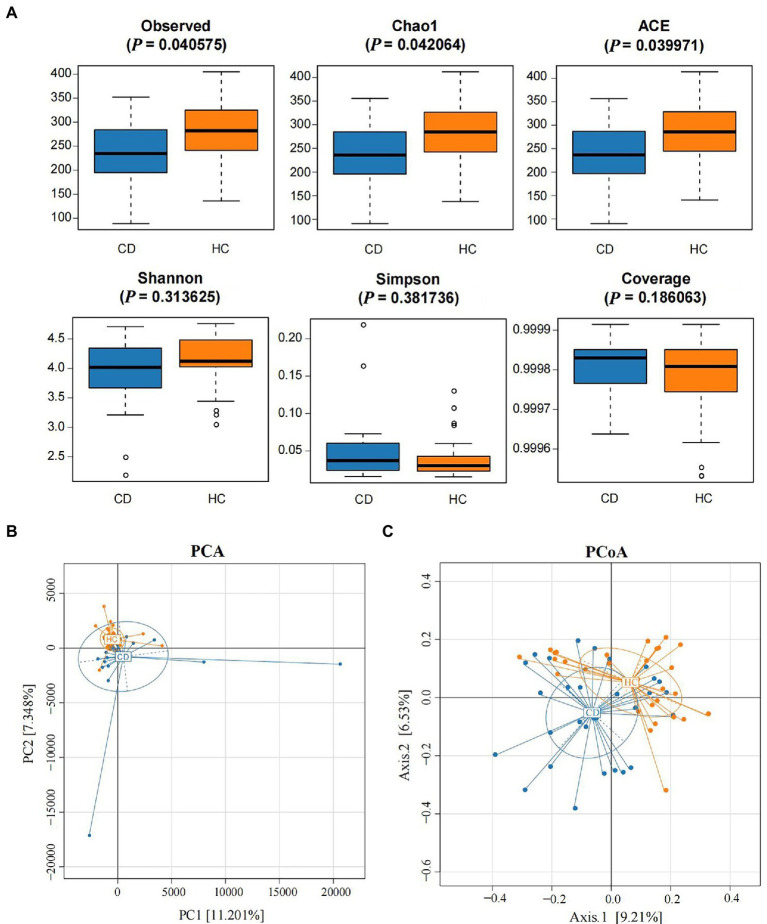
Alpha diversity analysis and beta diversity. **(A)** The comparison of gut microbiota alpha diversity between each group, including Observed species, Chao1, ACE, Shannon, Simpson, and Coverage; **(B)** Principal Component Analysis; **(C)** Principal Coordinate Analysis.

The abundance and diversity of species in each group were assessed by alpha diversity analysis. Alpha diversity analysis indices mainly included observed species, Chao 1, ACE, Shannon, Simpson, and Coverage. The results showed that the alpha diversity of gut microbiota in the CD group was lower than in the HC group. Among them, Observed, Chao 1, and ACE indicated that there were significant differences in microbial community richness between the CD and HC groups (*p* < 0.05), while other indexes showed no significant differences between the two groups (*p* > 0.05), as shown in Figure 2A.

PCA and PCoA analysis showed that there was significant separation of fecal microbial community structure between CD and HC groups, as shown in Figures 2B,C. ANOSIM based on UniFrac distances was calculated (R = 0.1038, *p* = 0.0005), further indicating significant differences in the bacterial communities among groups. There was no significant difference in the bacterial communities among different grades of Marsh (R = 0.0505, *p* = 0.2247) (Supplementary Figure S1).

**Figure 3:**
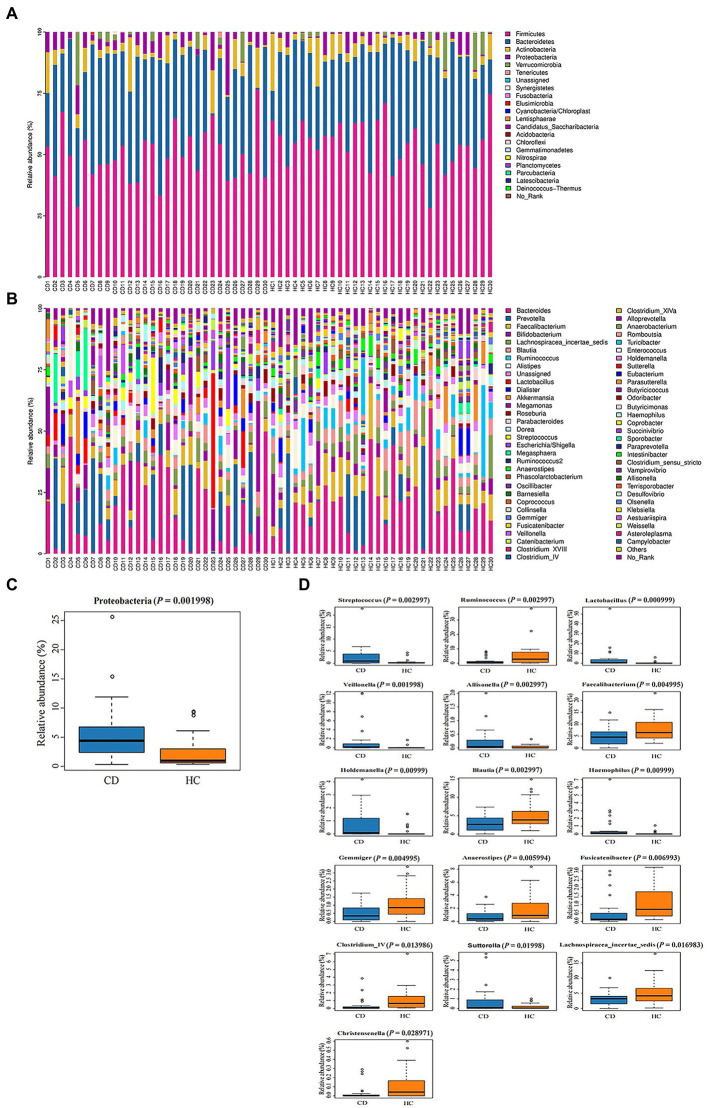
**(A)** Histogram of microbial community composition of each sample at the phylum level; **(B)** Histogram of microbial community composition of each sampl at the genus level; **(C)** Phylum level difference analysis; **(D)** Analysis of genus level differences.

The relative abundance of microorganisms at different classification levels was compared. At the phylum level, gut microbiota was mainly composed of *Firmicutes*, *Bacteroidetes* and *Actinobacteria* (Figure 3A). Metastats analysis showed that, at the phylum level (Figure 3C), the *Proteobacteria* abundance in the CD group was significantly higher than that in the HC group (5.53% vs. 2.58%, *Q* = 0.015). At the genus level, there were nine bacteria with differences in the CD group compared with the HC group (Figures 3B,D): the abundance of *Streptococcus, Lactobacillus, Veillonella,* and *Allisonella* communities in the CD group were significantly increased (Q < 0.05), while the abundance of *Ruminococcus, Faecalibacterium*, *Blautia*, *Gemmiger*, and *Anaerostipes* communities were significantly decreased (*Q* < 0.05). There were no significant difference in relative abundance of fecal bacterial communities composition at the genus level in different grades of Marsh (*Q* > 0.05) (Supplementary Tables S3–S5).

**Figure 4:**
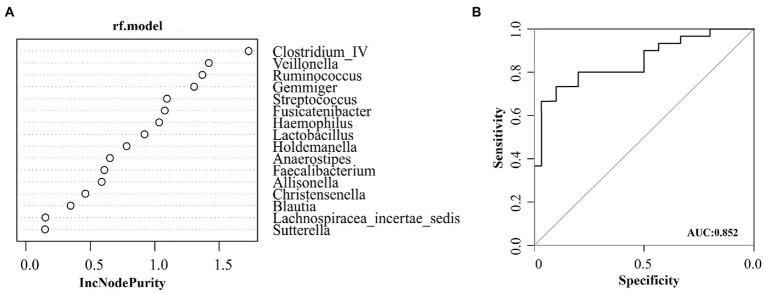
Based on species at genus level, the diagnostic models were constructed to distinguish CD patients from healthy controls. **(A)** Random forest model constructed in the CD and HC group; **(B)** ROC analysis for the top four species that can distinguish CD from HC.

Many different genera were screened out in the significance test of the difference between the two groups. To screen for species (biomarkers) that can distinguish CD disease status from HC, a diagnostic model based on genus level species was constructed. The AUC-random forests algorithm is used to determine an optimal random forest model that maximizes the AUC value of the ROC curve. In the validation queues of the CD and HC groups, four species were selected in order of importance where the AUC value was highest. The four species with the highest importance were *Clostridium_IV, Veillonella, Ruminococcus,* and *Gemmiger,* as shown in Figure 4A. The AUC was 0.85 (95% confidence interval [CI]: 0.754–0.951) (Figure 4B).

### Fecal metabolites analysis

**Figure 5:**
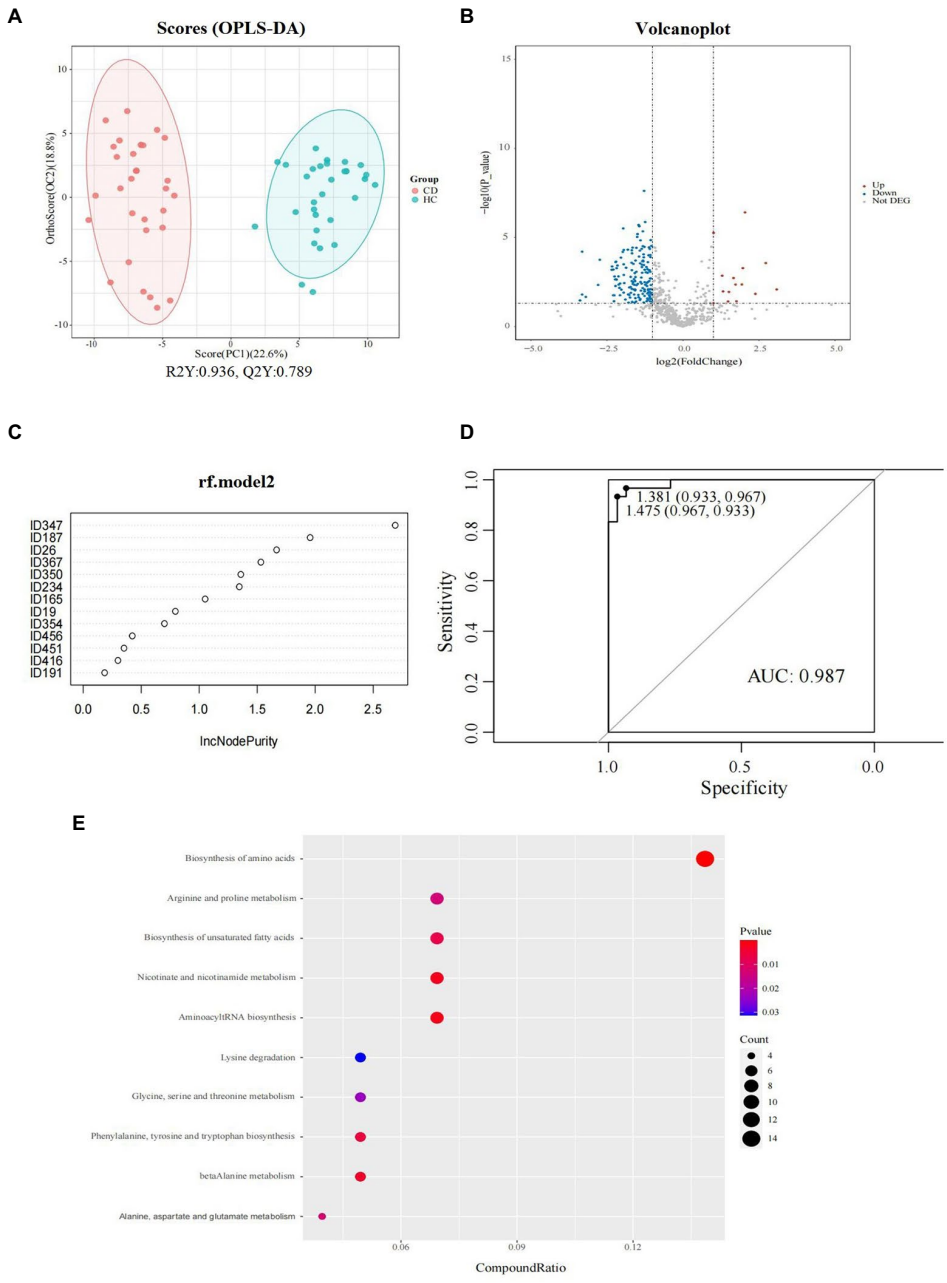
Fecal metabolomics analysis between groups in CD patients. **(A)** Orthogonal partial least squares discriminate analysis (OPLS-DA) score plots between the CD and HC groups; **(B)** Volcano plot demonstrated metabolites changes in CD compared with HC group; **(C)** Random forest model composed of metabolites was constructed to discriminate CD patients from healthy controls. **(D)** ROC analysis for the top 6 metabolites that can distinguish CD from HC. **(E)** The bubble plot of KEGG analysis. Bubble charts show the top 10 metabolic pathways that the differentially expressed metabolites enriched in between CD and HC groups.

The results of OPLS-DA (Figure 5A) showed that there was a difference in the fecal metabolite spectrum between the CD and HC groups (R2Y = 0.936, Q2Y = 0.789), indicating that the fecal metabolites of CD were transformed, and the difference between the two groups could well be reflected at the metabolic level. Using VIP >1 and *p* < 0.05 of the first principal component of OPLS-DA model as screening conditions, 222 annotatable differential metabolites were screened from CD and HC groups, including 25 up-regulated and 197 down-regulated metabolites (Figure 5B). The screening conditions were further adjusted, and the screening conditions were set as VIP >2.0 and *p* < 0.05. Finally, a total of 13 metabolites with significant differences were screened between CD and HC groups (Table 2). PLS-DA models have poor interpretability and predictability in different Marsh grades (Supplementary Figure S2; Table S6).

**Table 2:** Differential Fecal metabolites between groups in CD patients (VIP > 2 and *P* < 0.05).

**No.**	**ID**	**Metabolite**	**VIP**	**FC**	**Metabolite changes**	**AUC**	**95%CI**
1	ID347	D-Alanyl-D-serine	2.605	2.041	Up	0.953	0.906–1.000
2	ID367	Formiminoglutamic acid	2.206	1.492	Down	0.918	0.847–0.988
3	ID187	Acetylcholine	2.187	1.503	Down	0.873	0.778–0.969
4	ID19	Docosapentaenoic acid (22n-3)	2.187	2.789	Down	0.892	0.808–0.977
5	ID354	S-Methyl-L-methionine	2.130	1.317	Down	0.879	0.794–0.964
6	ID165	Epiandrosterone	2.122	2.182	Down	0.873	0.783–0.964
7	ID26	Coronopilin	2.105	2.038	Down	0.891	0.800–0.982
8	ID451	(+_-)-5-[(tert-Butylamino)-2′-hydroxypropoxy]-3,4-dihydro-1(2H)-naphthalenone	2.069	1.655	Down	0.890	0.806–0.975
9	ID416	Traumatic Acid	2.062	2.733	Down	0.873	0.788–0.958
10	ID234	LysoPA(16_0_0_0)	2.022	1.276	Down	0.910	0.833–0.987
11	ID456	Pentyl octanoate	2.021	2.198	Down	0.881	0.798–0.964
12	ID350	N (6)-Methyllysine	2.011	1.130	Down	0.882	0.795–0.969
13	ID191	6-Hydroxynicotinic acid	2.002	1.456	Down	0.848	0.751–0.945

VIP, variable importance in the projection; FC, fold change; AUC, area under the curve; CI, confidence interval.

Six metabolites were screened as potential biomarkers by random forest model, including: acetylcholine, D-alanyl-D-serine, coronopilin, formiminoglutamic acid, N (6)-methyllysine, lyso PA (16_0_0_0). The AUC was 0.97 (95% CI: 0.967–1) (Figures 5C,D).

The metabolic pathway enrichment results were obtained based on KEGG pathway database. The results indicated that the different metabolites enriched in metabolic pathways, as follows: biosynthesis of amino acids; arginine and proline metabolism; biosynthesis of unsaturated fatty acids; nicotinate and nicotinamide metabolism; aminoacyl tRNA biosynthesis; lysine degradation; glycine, serine and threonine metabolism; phenylalanine, tyrosine, and tryptophan biosynthesis; beta alanine metabolism; and alanine, aspartate, and glutamate metabolism (Figure 5E). Among these pathways, the biosynthesis of amino acids contributed the most to the metabolic differences.

### Correlation analysis of CD-related differential microorganisms and metabolites

**Figure 6:**
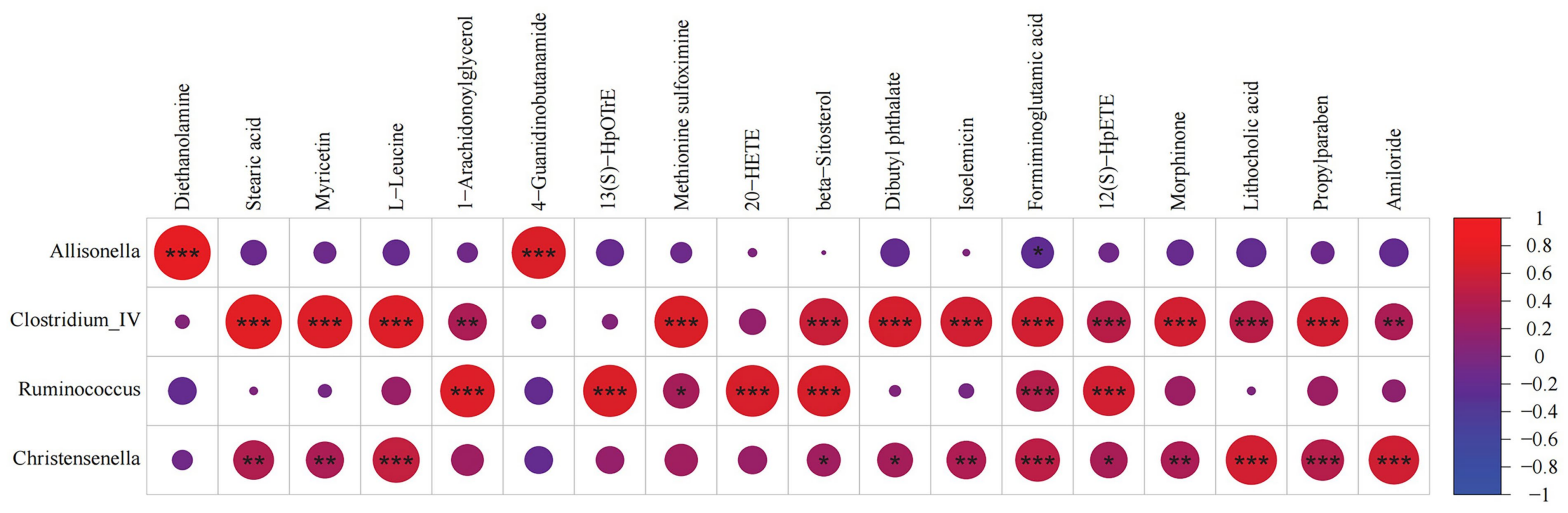
Correlation analysis between fecal microorganisms and metabolites. A correlation matrix was drawn correlation analysis of differential bacteria and metabolites. * 0.01 ≤ *p* < 0.05, ** 0.001 ≤ *p* < 0.01, *** 0.0001 ≤ *p* < 0.001.

Pearson correlation analysis was used to analyze the relationship between fecal metabolites and microbiomes. We found that *Allisonella, Clostridium_IV, Ruminococcus,* and *Christensenella* were strongly correlated with differential metabolites, as shown in Figure 6. *Allisonella* was positively correlated with diethanolamine and 4-guanidinobutanamide. *Clostridium_IV* was positively correlated with stearic acid, myricetin, L-leucine, methionine sulfoximine, beta-sitosterol, dibutyl phthalate, isoelemicin, formiminoglutamic acid, 12(S)-HpETE, morphinone, lithocholic acid, and propylparaben. *Ruminococcus* was positively correlated with 1-arachidonoylglycerol, 13(S)-HpOTrE, 20-HETE, beta-sitosterolm, formiminoglutamic acid, and 12(S)-HpETE. *Christensenella* was positively correlated with L-leucine, formiminoglutamic acid, lithocholic acid, propylparaben, and amiloride. These correlations suggest that the changes of gut microbiota structure and metabolites are closely related to host metabolism.

## Discussion

Advances in the field of microbial research have established a clear link between the gut microbiome and human health (Weir et al., 2021). There is increasing evidence that gut microbiota and their metabolites play an important role in the development of CD, since they are closely related to dietary habits, genetic background, and geographical factors. The incidence of CD in China, for instance, has ethnic and regional differences, but there is a lack of research on gut microbiota related to CD. Xinjiang is located in the Northwest of China, which is a multi-ethnic region with unique geographical environment, genetic background and population composition. Therefore, the conclusions drawn by relevant studies carried out in other countries and regions may not be completely applicable to the population in this region. The purpose of this study is to analyze the fecal microbial composition and metabolome characteristics in patients diagnosed with CD in Northwest China. The results emphasize that the imbalance of gut microbiota resulting in metabolic disorders is an important factor in the pathogenesis of CD, providing some new clues the use of gut microbiota and fecal metabolites as biomarkers to screen or diagnose CD, and providing intervention targets its future treatment.

In the past, CD was considered rare in China; however, a growing number of patients have been diagnosed in recent years. Previous research showed that the seroprevalence of CD in the general Chinese population was 0.27% (Zhou et al., 2021), while the prevalence of positive results from serologic testing in Xinjiang is 1.27% (Zhou et al., 2020); this is higher than the reported national average and closer to the pooled global average (Singh et al., 2018). Gluten is recognized as the main external environmental trigger of CD (Serena et al., 2019b); andhe consumption of gluten-containing cereals in Xinjiang is very high. According to the Xinjiang Statistic Year Book data (2021), the yearly cereal intake per person was 151.81 kg in total: 129.27 kg/person/year in urban Xinjiang and 171.89 kg/person/year in rural Xinjiang. Therefore, high wheat consumption is a critical environmental risk factor for the occurrence of CD in this area. Gut microbiota have host genetic relevance. Some studies have shown that HLA-DQ genotype may affect the first generation of gut colonizers, and this change in gut microbiota increases the risk of CD (Olivares et al., 2015; Valitutti et al., 2019). Data from a meta-analysis data indicate that the frequency of the DQB1*0201 allele frequency is particularly high (22.04%) in the populations (Yuan et al., 2013). Zhou et al. (2020) described the frequency of the HLA-DQ2 and-DQ8 haplotypes among four major ethnic groups in Xinjiang: it was highest in the Uyghur (52.1%), followed by the Hui (44.4%), the Kazakh (40.0%) and the Han (39.4%). Therefore, the genetic susceptibility for CD is highly prevalent in the Xinjiang region. These factors also lead to the special dietary habits of its residents, diet plays an important role in regulating the composition of human gut microbiota and metabolic activities, and dietary factors in early life may largely determine the risk of future health or disease (Chen et al., 2018; Valitutti et al., 2019; Trakman et al., 2022). The intake of carbohydrates and fat in the dietary structure of Xinjiang is high, while the intake of micronutrients, dietary fiber and high-quality protein is low (Yin et al., 2020; Tao et al., 2022). Because of its unique geographical environment, genetic background, population composition, and dietary culture, the fecal microbial composition and metabolome characteristics of patients with CD was unique in this region.

In this study, *Firmicutes* and *Bacteroidetes* were the dominant genera between the two groups, similar to other human gut microbiota studies (Verdu et al., 2015; Kumar et al., 2018; Valitutti et al., 2019), while *Proteobacteria* community abundance in the CD group was significantly higher than in the HC group, which may contribute to the development of the disease. In rat models, the combined action of gliadin and *Proteobacteria* member *Shigella* has been proved to enhance the destruction of the intestinal epithelial barrier (Cinova et al., 2011), possibly because *Proteobacteria* dominated bacteria and toxins are more likely to penetrate the intestinal mucus layer (Jakobsson et al., 2015). Wacklin et al. (2013) found that increased *Proteobacteria* colonization was associated with the common gastrointestinal manifestations of CD. In addition, *Proteobacteria* with adhesive and invasive properties are thought to promote inflammatory responses, leading to inflammatory bowel disease in genetically or immunologically susceptible populations (Mukhopadhya et al., 2012). Therefore, this confirms that most individuals share a common microbiome with inter-individual variability, which can be explained by factors such as diet, geography, and ethnicity.

Current studies show that the change of microbiota in CD patients is mainly manifested by the increase of Gram-negative bacteria and the decrease of Gram-positive bacteria (Girbovan et al., 2017). At the genus level, fecal samples of CD in the region showed a significant decrease in the numbers of *Firmicutes* (*Ruminococcus, Faecalibacterium, Blautia, Gemmiger, and Anaerostipes*), which are all Gram-positive bacteria. There was also a higher abundance of *Proteobacteria* (*Holdemanella, Haemophilus, Sutterella*), *Bacteroidetes* (*Veillonella, Allisonella*), and *Firmicutes* (*Streptococcus, Lactobacillus*), which are mainly Gram-negative bacteria, indicating the imbalance of CD fecal microbiota. Among them, *Streptococcus*, *Haemophilus and Veronococcus* are all opportunistic pathogens, which mainly colonize and infect human beings and cause many diseases (Nørskov-Lauritsen, 2014; Andam and Hanage, 2015; Diricks et al., 2022). Moreover, several cohort studies have shown that different gastrointestinal infections increase the risk of CD, with viral and bacterial factors being involved in the pathophysiology of the disease (Caminero and Verdu, 2019). Previous studies have found that the incidence of *Streptococcus* significantly increased in the saliva and tissues of CD patients (Nistal et al., 2012a, 2016; Iaffaldano et al., 2018; Serena et al., 2019a; Zhan et al., 2022), and similar results were found in the stool samples of this study. Multiple studies (Ludvigsson et al., 2008; Thomas et al., 2008; Tjernberg et al., 2020) have shown an increased risk of invasive pneumococcal disease in CD, and the European CD guidelines recommend that CD patients are vaccinated with the *Streptococcus pneumoniae* vaccine (Al-Toma et al., 2019). Furthermore, *Veroniella* is a proinflammatory taxon that increases in patients with inflammatory bowel disease and irritable bowel syndrome (Shukla et al., 2015; Ruigrok et al., 2021) and promotes the expression of several inflammatory cytokines (TNF-α, IL-6, and IL-1β) in the colon (Shukla et al., 2015). It is also increased in stool samples from patients with CD or refractory CD (Garcia-Mazcorro et al., 2018b; Panelli et al., 2020). In general, the main features are increased abundance of potentially pathogenic Gram-negative bacteria, especially highly pathogenic gut bacteria, the activation of proinflammatory pathways, and the induced secretion of Th1 type proinflammatory cytokines (De Palma et al., 2010). In addition, most reports show that compared with healthy people, patients with CD have an imbalance of *Lactobacillus*, which shows that the diversity and abundance of probiotics are reduced (Girbovan et al., 2017; Valitutti et al., 2019; Serena et al., 2019a; Francavilla et al., 2020). Interestingly, we did not find these changes, and Nistal et al., 2012b also found similar results, which may be because the presence of *Lactobacillus* in the intestines is affected by dietary intake (Graf et al., 2015). People in Xinjiang have a high intake of fermented dairy products in their diet, and CD patients may have spontaneous excessive intake due to gastrointestinal symptoms. Therefore, the people in Northwest China have unique gut microbiota characteristics, and *Lactobacillus* as a supplement may not improve CD in this region.

Despite inflammation in CeD being localized to the small intestine, studies in both pediatric and adult populations have frequently employed fecal analysis to investigate the microbiota due to ease of sampling. However, functional heterogeneity of each gastrointestinal tract segment gives rise to regional differences in gut microbial populations (Martinez-Guryn et al., 2019). The regional tropism of gastrointestinal tract microbiota can be a determinant of specific diseases. A recent study found that (Constante et al., 2022) microbiota clustering based on Marsh grades within the duodenal mucosa, but not within aspirate or fecal samples, indicated the duodenal microbiota is altered in association with intestinal damage, also suggests that the result based only on fecal analysis must be interpreted with caution. Our study also found that the fecal microbiota and metabolome were not associated with intestinal injury. Therefore, the severity of intestinal damage in CD may mainly affect the change of the duodenal mucosal microbiota. Although this is a fair argument to propose, it is possible that events in the large bowel influence disease pathogenesis upstream along the gastrointestinal tract. Irrespective of their primary role in CD pathogenesis, the disease-specific microbial signature identified here might be used as another adjutant, noninvasive biomarker to screen for CD.

Gut microbiota regulate a series of key metabolic functions in the body, and its imbalance is an important factor in regulating host metabolic disorders (Pascale et al., 2018). In a recent study, aspirates from the small intestine of CD donors were poured into the stomachs of mice. The intestinal contents from mice colonized with CD microbiota exhibited altered gluten metabolism, suggesting that proteolytic function is transmitted by the microbiota associated with an inflamed celiac duodenum, and further supporting the role of microbial metabolism in CD (Constante et al., 2022). The fecal metabolome can serve as an intermediate phenotype mediating host-microbiome interactions. It could potentially be a new tool for exploring the links between microbiome composition, host phenotype, and heritable complex traits (Zierer et al., 2018). To the best of our knowledge, there are few studies using LC–MS/MS untargeted metabolomics to investigate fecal metabolome changes in adult CD subjects. There is also a lack of joint analysis of relevant microorganisms and metabolites in patients with CD. In this study, we used 16S rDNA and metabolomics sequencing to reveal changes in gut microbiome composition and underlying mechanisms of gut microbiome mediated metabolic dysfunction in CD patients in Northwest China. The significant difference in fecal metabolite levels between CD and HC groups suggest that CD can cause changes in fecal related terminal metabolites. In our study, 222 fecal metabolites that were differentially regulated between sample sets were identified. Finally, 13 metabolites with significant differences between the CD and HC groups were screened using VIP >2.0 and *p* < 0.05 as screening conditions. Mainly, the levels of amino acids and their derivatives (metabolites, steroids, lipids, choline, oxidized derivatives of unsaturated fatty acids, esters, and niacin metabolites) were decreased in the CD group. According to pathway enrichment analysis, they are mainly involved in the metabolic pathways of biosynthesis and metabolism of amino acids, biosynthesis of unsaturated fatty acids, nicotinate and nicotinamide metabolism, and in aminoacyl tRNA biosynthesis. The decreased levels of choline (Acetylcholine), methionine (S-methyl-L-methionine, a derivative of methionine) and lipids (especially lysoPA (16_0_0_0), docosapentaenoic acid (22 N-3)) indicated the deficiency of one-carbon metabolism (OCM) in CD patients. Martín-Masot et al. (2020) were the first to describe metabolic deficiencies in OCM in patients with CD. OCM is a network of biochemical reactions in which one-carbon units are transferred to various biosynthetic pathways; folic acid and methionine cycles are two important components of this network (Newman and Maddocks, 2017; Pan et al., 2021; Lionaki et al., 2022). They are functionally used for biosynthesis of phospholipids, amino acids and DNA, as well as methylation of proteins, RNA, and DNA. CD mainly causes progressive villous atrophy, crypt hyperplasia and proliferation of epithelial lymphocytes in small intestinal mucosa, affecting the absorption of trace elements such as folic acid and vitamin B_12_. This results in malnutrition (Balaban et al., 2019) and damage to energy generation pathways (Sharma et al., 2015; Upadhyay et al., 2020), while a close dependence between the regulatory pathways of energy and OCM, as well as the balance between input and output, remains (Luciano-Mateo et al., 2017). Therefore, the deficiency of OCM in CD patients may be related to energy deficiency caused by malabsorption. These findings will provide new translational opportunities for CD dietary interventions, drug development, and biomarker identification.

Previous studies have found higher levels of short-chain fatty acids (SCFAs) in both untreated and treated patients with CD than in healthy controls (Nistal et al., 2012b; Tjellström et al., 2013; Caminero et al., 2015)—these findings usually involve pediatric patients. However, in this study, there was no significant difference in the content and composition of SCFAs between adult CD patients and HCs, which is consistent with the results of Niccolai et al.’s (2019) study. They considered that age may affect the composition of SCFAs, and the disease-specific mechanism involving gut microbiota is still unclear. In the gastrointestinal tract, tryptophan can be metabolized by the gut microbiota into ligands of the aryl hydrocarbon receptor (AhR), which contributes to intestinal homeostasis (Roager and Licht, 2018). Lamas et al. (2020) identified a potential pathogenic mechanism related to the impaired production of AhR ligands by the gut microbiota in CD. A recent study has been found that tryptophan metabolism in patients with potential CD is lower than in both CD and HC groups. The altered metabolic profile of patients with potential CD suggested that gluten intolerance was evident at the metabolic level before intestinal damage (Upadhyay et al., 2022). This study found that tryptophan derivative N-Acetyl-D-tryptophan in stool metabolites of CD patients was downregulated. Therefore, based on the aforementioned evidence, a tryptophan-enriched diet may be an effective adjuvant treatment for CD.

We further explored the potential use of gut microbiota related metabolites in the noninvasive diagnosis of CD. The gut microbiota of four genera (*Clostridium_IV, Veillonella, Ruminococcus, and Gemmiger*) can distinguish CD from HC, with an AUC of 0.85. The model based on six metabolites (D-alanyl-D-serine, acetylcholine, coronopilin, formiminoglutamic acid, N(6)-methyllysine, and lysoPA) performed better than microbial characteristics, with an AUC of 0.98. The current diagnosis of CD is based on a combination of features, including CD specific serum antibodies (anti-tTG antibody, anti-endomysial antibody, or anti-DGP antibody) and the presence of villous atrophy assessed by intestinal mucosal biopsy (Bai and Ciacci, 2017; Al-Toma et al., 2019). However, poor or inadequate biopsy specimens and patchy mucosal damage may increase the risk of false positive or false negative results (Pais et al., 2008; Ravelli et al., 2010; Mubarak et al., 2011). Therefore, we need more accurate noninvasive biomarkers to complement traditional serological tests to diagnose CD.

In this study, the gut microbiota and metabolites were linked with a multi-omics integration method. Correlation analysis showed that the decrease of Gram-positive bacteria, such as *Firmicutes* (*Clostridium_IV, Ruminococcus Christensenella*), was closely related to amino acid metabolism, esters, alcohols and other organic compounds. Overall, these results suggest a significant interaction between gut microbiota and metabolites that may influence the occurrence of CD. By studying the correlation between some metabolites and microbial communities, it may eventually help to manipulate the function of microbial communities in CD therapeutically (McHardy et al., 2013; Gundamaraju et al., 2017).

This study has some limitations. Most importantly, it has a relatively small sample size, and different diagnostic models based on microbial composition and metabolites may limit the generalization of the results, requiring external validation through larger samples and multi-center trials. Nevertheless, confounding factors can be effectively controlled by recruiting age-, sex-and ethnic-matched healthy subjects. Moreover, this was a case–control study. Although our data proposed the functional association between microbiome, metabolome, and disease, cause and effect could not be distinguished, and the mechanism of functional association needs to be further explored.

In conclusion, studying the relationship between human gut microbiota and CD in Northwest China has highlighted some unique microbiota and metabolic characteristics and opened a new way for researchers to explore the potential beneficial effects of supplementing specific nutrients. This study provides an experimental basis for formulating potential diagnostic and therapeutic targets in the future. These results should encourage more research on CD in China.

## Data availability statement

The datasets presented in this study can be found in online repositories. The names of the repository/repositories and accession number(s) can be found below: NCBI-PRJNA890948.

## Ethics statement

The studies involving human participants were reviewed and approved by the consent of the ethics committee of the people’s Hospital of Xinjiang Uygur Autonomous Region (approval number: KY202203110 67). The patients/participants provided their written informed consent to participate in this study.

## Author contributions

TS and GF: conceptualization. TS: writing original draft. YF and AA: data curation. WL and TL: software. HL and MW: formal analysis. ZL and JL: methodology. AM and HJ: project administration. GF: supervision. All authors contributed to the article and approved the submitted version.

## Funding

This work was supported by the National Natural Science Foundation of China (82260116), the Natural Science Foundation of Xinjiang Province (2021D01C149), and the Hospital Project of People’s Hospital of Xinjiang Uygur Autonomous Region (20200406).

## Conflict of interest

The authors declare that the research was conducted in the absence of any commercial or financial relationships that could be construed as a potential conflict of interest.

## Publisher’s note

All claims expressed in this article are solely those of the authors and do not necessarily represent those of their affiliated organizations, or those of the publisher, the editors and the reviewers. Any product that may be evaluated in this article, or claim that may be made by its manufacturer, is not guaranteed or endorsed by the publisher.

## Footnotes

^1^ http://www.hmdb.ca

^2^ http://www.massbank.jp/

^3^ http://www.Lipidmaps.org

^4^ https://www.mzcloud.org

^5^ http://www.Genome.jp/kegg/

^6^ http://www.genome.jp/kegg/

^7^ https://www.metaboanalyst.ca/
