# Supplementary material for: Characteristics of gut microbiota and fecal metabolomes in patients with celiac disease in Northwest China
Source: Front Microbiol. 2022 Nov 28;13:1020977. doi: 10.3389/fmicb.2022.1020977 (PMC9742481; doi:10.3389/fmicb.2022.1020977)
Supplement: Supplementary file 1 [file Data_Sheet_1.PDF]

Supplementary Material

1. Supplementary Figures and Tables

1.1 Supplementary Figures

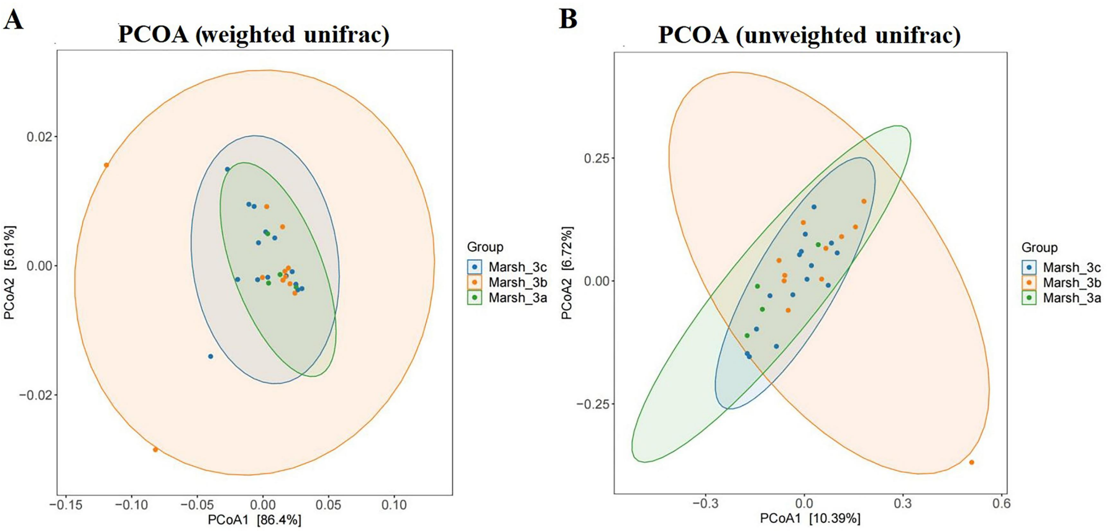

Figure. S1 Beta diversity analysis among different Marsh score. (A, B) Principal coordinates analysis (PCoA) analysis based on weighted unifrac distance and unweighted unifrac distance.

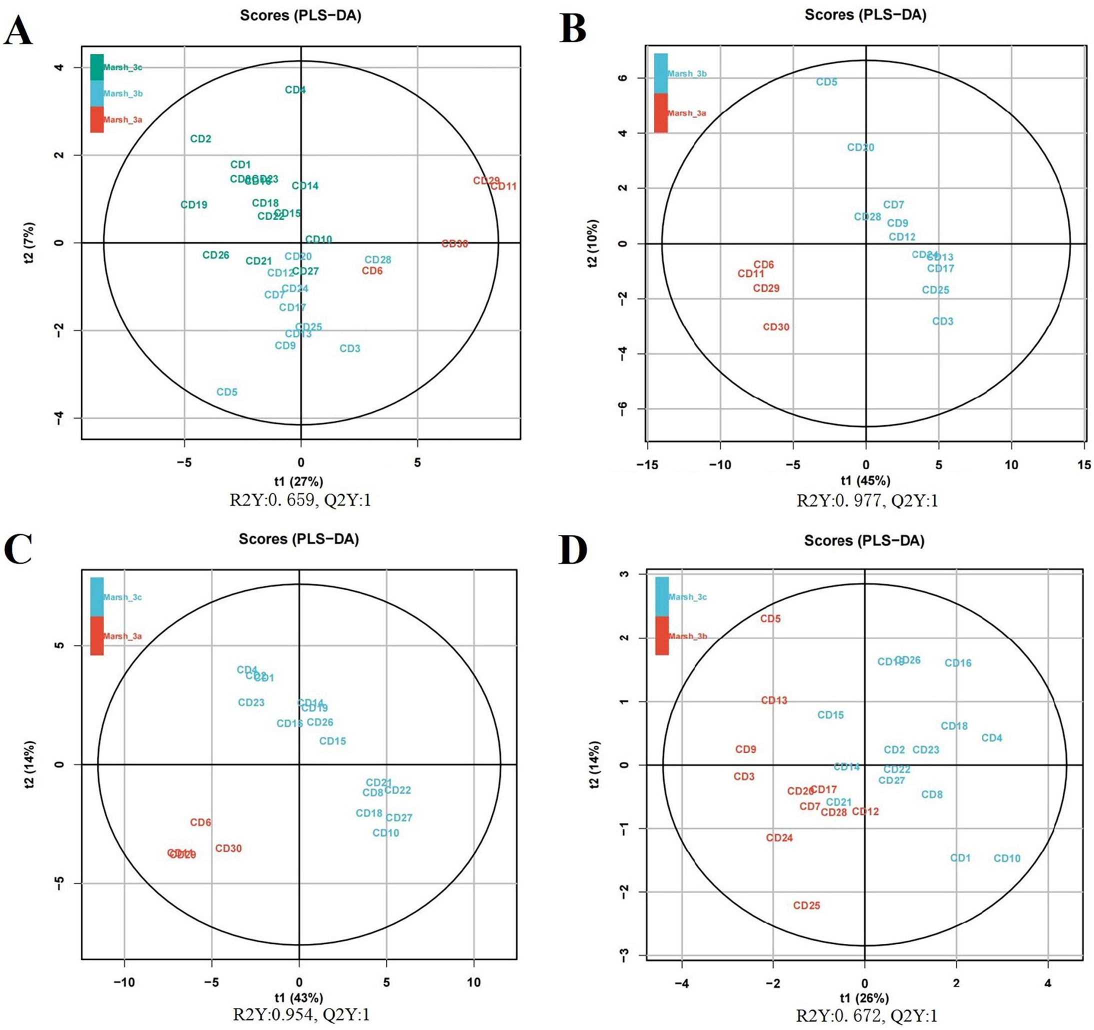

Figure. S2 (A, B, C, D) are the Scatter plot of partial least squares discriminant analysis model (PLS-DA) scores in different Marsh grades, respectively. The abscissa is the score of the sample on the first principal component, and the ordinate is the score of the sample on the second principal component. R2Y represents the interpretation rate of the model, Q2Y is used to evaluate the predictive ability of the PLS-DA model, and when R2Y is greater than Q2Y, the model is well established.

1.2 Supplementary Tables

Table S1. Demographic characteristics of patients with CD

Sample NO.	Sex	Age (years)	Ethnic	Serology	Marsh score
1	female	30	Kazakh	+ tTG	3c
2	female	50	Uyghur	+ tTG	3c
3	male	20	Uyghur	+ tTG	3b
4	female	21	Kazakh	+ tTG	3c
5	female	38	Uyghur	+ tTG	3b
6	female	36	Kazakh	+ tTG	3a
7	male	19	Uyghur	+ tTG	3b
8	male	58	Uyghur	+ tTG	3c
9	male	44	Kazakh	+ tTG	3b
10	female	46	Kazakh	+ tTG	3c
11	female	65	Han	+ tTG	3a
12	female	33	Kazakh	+ tTG	3b
13	female	24	Uyghur	+ tTG	3b
14	female	55	Uyghur	+ tTG	3c
15	female	50	Kazakh	+ tTG	3c
16	female	39	Uyghur	+ tTG	3c
17	male	47	Han	+ tTG	3b
18	female	45	Kazakh	+ tTG	3c
19	female	40	Kazakh	+ tTG	3c
20	female	20	Uyghur	+ tTG	3b
21	male	54	Uyghur	+ tTG	3c
22	female	56	Kazakh	+ tTG	3c
23	female	44	Kazakh	+ tTG	3c
24	female	43	Uyghur	+ tTG	3b
25	male	51	Kazakh	+ tTG	3b
26	female	40	Uyghur	+ tTG	3c
27	female	40	Uyghur	+ tTG	3c
28	female	41	Uyghur	+ tTG	3b
29	female	26	Han	+ tTG	3a
30	female	51	Han	+ tTG	3a

Table S2. Demographic characteristics of controls

Sample NO.	Sex	Age (years)	Ethnic	Serology	Marsh score
1	female	32	Kazakh	- tTG	–
2	female	49	Uyghur	- tTG	–
3	male	22	Uyghur	- tTG	–
4	female	24	Kazakh	- tTG	–
5	female	39	Uyghur	- tTG	–
6	female	40	Kazakh	- tTG	–
7	male	19	Uyghur	- tTG	–
8	male	56	Uyghur	- tTG	–
9	male	46	Kazakh	- tTG	–
10	female	43	Kazakh	- tTG	–
11	female	60	Han	- tTG	–
12	female	30	Kazakh	- tTG	–
13	female	23	Uyghur	- tTG	–
14	female	53	Uyghur	- tTG	–
15	female	43	Kazakh	- tTG	–
16	female	36	Uyghur	- tTG	–
17	male	47	Han	- tTG	–
18	female	46	Kazakh	- tTG	–
19	female	39	Kazakh	- tTG	–
20	female	21	Uyghur	- tTG	–
21	male	49	Uyghur	- tTG	–
22	female	54	Kazakh	- tTG	–
23	female	39	Kazakh	- tTG	–
24	female	41	Uyghur	- tTG	–
25	male	50	Kazakh	- tTG	–
26	female	37	Uyghur	- tTG	–
27	female	38	Uyghur	- tTG	–
28	female	41	Uyghur	- tTG	–
29	female	26	Han	- tTG	–
30	female	52	Han	- tTG	–

–, not collected from controls.

Table S3. Difference in the relative abundance (> 1%) of fecal bacteria community composition at the genus level in Marsh3a and 3b groups

Items	Marsh3a	Marsh3b	*P*-value	*Q*-value
Oscillibacter	0.04±0.03	1.16±0.30	0.002	0.127
Gemmiger	0.08±0.05	0.65±0.15	0.003	0.127
Odoribacter	0.06±0.06	0.71±0.19	0.004	0.127
Barnesiella	0±0	1.46±0.48	0.007	0.141
Bilophila	0.02±0.02	0.15±0.04	0.010	0.173
Holdemanella	0±0	0.80±0.31	0.018	0.195
Vampirovibrio	0±0	0.27±0.11	0.023	0.222
Butyricimonas	0.01±0.01	0.44±0.18	0.031	0.280
Alistipes	0.38±0.37	3.64±1.38	0.034	0.283
Paraprevotella	0±0	0.19±0.09	0.043	0.333
Clostridium_XlVb	0.03±0.02	0.23±0.09	0.046	0.333

Data presented as mean±standard deviation

Table S4. Difference in the relative abundance (> 1%) of fecal bacteria community composition at the genus level in Marsh3a and 3c groups

Items	Marsh3a	Marsh3c	*P*-value	*Q*-value
Barnesiella	0±0	1.08±0.30	0.003	0.166
Clostridium_XlVb	0.03±0.05	0.18±0.04	0.004	0.166
Gemmiger	0.08±0.05	0.55±0.14	0.007	0.166
Holdemanella	0±0	1.02±0.33	0.007	0.166
Butyricimonas	0.01±0.01	0.44±0.14	0.010	0.166
Oscillibacter	0.03±0.02	0.84±0.32	0.024	0.228

Data presented as mean±standard deviation

Table S5. Difference in the relative abundance (> 1%) of fecal bacteria community composition at the genus level in Marsh3b and 3c groups

Items	Marsh3b	Marsh3c	*P*-value	*Q*-value
Succinivibrio	0.54±0.54	0±0	0.001	0.019
Veillonella	0.13±0.05	1.93±0.87	0.005	0.060
Lactobacillus	0.84±0.43	6.80±0.04	0.018	0.171
Haemophilus	0.09±0.02	0.99±0.48	0.028	0.235
Campylobacter	0±0	0.15±0.14	0.049	0.357

Data presented as mean±standard deviation

Table S6. Metabolite differential screening results in different Marsh grades

Group	Num of Total Sig	Num of Sig Up	Num of Sig down	R2Y	Q2Y
Marsh_3a vs Marsh_3b	57	5	52	0.977	1
Marsh_3a vs Marsh_3c	55	8	47	0.954	1
Marsh_3b vs Marsh_3c	11	8	3	0.672	1
Marsh_3a vs Marsh_3b vs Marsh_3c	42	–	–	0.659	1

Num of Sig Up, the total number of metabolites significantly upregulated; Num of Sig down, the total number of metabolites significantly downregulated; R2Y represents the interpretation rate of the model, Q2Y is used to evaluate the predictive ability of the PLS-DA model, and when R2Y is greater than Q2Y, the model is well established.
